# Chest CT imaging for differentiating normal, PRISm, and COPD in comparison with pulmonary function tests

**DOI:** 10.1007/s11547-025-02061-4

**Published:** 2025-08-21

**Authors:** Zongjing Ma, Yingli Sun, Zhuangxuan Ma, Ling Zhang, Fanzhi Cheng, Haihong Ma, Liang Jin, Ming Li

**Affiliations:** 1 Department of Radiology, Huadong Hospital, Fudan University, Shanghai, China; 2 Department of Radiology, Fudan University Shanghai Cancer Centre, Shanghai, China; 3 Kashi Prefecture Second People′s Hospital, Xinjiang, China

**Keywords:** Chronic obstructive pulmonary disease, Preserved ratio impaired spirometry, Radiomics, Multinomial logistic regression

## Abstract

**Background:**

Preserved ratio impaired spirometry (PRISm) and chronic obstructive pulmonary disease (COPD) are progressive respiratory disorders associated with accelerated pulmonary function decline and systemic comorbidities. This multicenter study aimed to develop a three-category classification model that integrates clinical variables with thoracic computed tomography (CT) radiomics to distinguish normal pulmonary function, PRISm, and COPD.

**Methods:**

A total of 1018 participants from three centers (A, B, C) who underwent chest CT and pulmonary function tests (PFTs) within a 2-week interval were retrospectively analyzed. After applying inclusion and exclusion criteria, 797 individuals were included for analysis (Center A: 667 [training/internal test = 534:133]; Centers B, C: 130 external test). CT images were preprocessed via resampling and intensity normalization, followed by semi-automated segmentation of the airway tree and whole lung parenchyma using Mimics Research. PyRadiomics extracted 2436 radiomic features (1218 per region). Feature selection combined maximum relevance minimum redundancy with least absolute shrinkage and selection operator regression, employing tenfold cross-validation. Five models were developed using multinomial logistic regression: (1) clinical model, (2) airway model, (3) lung model, (4) airway fusion model, and (5) lung fusion model. Performance metrics included accuracy, sensitivity, specificity, positive predictive value, negative predictive value, and the area under the receiver operating characteristic curve (AUC), with DeLong tests comparing model efficacy.

**Results:**

35 airway tree and 48 lung radiomic features were ultimately selected. The best performing model was the lung fusion model, which integrated three clinical predictors (age, gender, and BMI) with selected lung radiomic features. In external test set, it achieved superior performance with AUCs of 0.939 (95% CI 0.898–0.979) for PFT-normal, 0.830 (0.758–0.902) for PRISm, and 0.904 (0.841–0.966) for COPD, with an overall accuracy of 83.59%. DeLong tests indicated that across all three datasets, the lung fusion model outperformed the other four models.

**Conclusion:**

Combining age, gender, BMI, and lung radiomic features significantly improves detection of PRISm and COPD compared to alternative models. These findings underscore the potential of CT-based radiomics for the early identification and risk stratification of abnormal pulmonary function.

**Supplementary Information:**

The online version contains supplementary material available at 10.1007/s11547-025-02061-4.

## Background

Chronic obstructive pulmonary disease (COPD) is a chronic inflammatory condition characterized by persistent airflow limitation, with high prevalence, mortality, and economic burden, making it the third leading cause of death worldwide [1]. Given the disease’s progressive nature, early intervention is crucial for slowing the decline in pulmonary function and improving long-term outcomes. Notably, some patients with abnormal pulmonary function do not meet the diagnostic criteria for typical COPD but present with preserved ratio impaired spirometry (PRISm), defined by a reduced forced expiratory volume in 1 s (FEV₁) and a FEV₁/forced vital capacity (FVC) ratio ≥ 0.70 [2]. PRISm exhibits marked heterogeneity in its inflammatory biomarker profiles, imaging characteristics, and clinical trajectories, with some cases reverting to normal function while others progress to COPD [3–5]. Emerging evidence suggests that early interventions, including oxygen therapy and bronchodilators, can effectively delay the progression of PRISm, underscoring the clinical importance of accurate phenotypic identification [6–8].

Chest CT in PRISm patients often reveals airway wall thickening, which is strongly linked to small airway dysfunction [9, 10]. Quantitative chest CT analysis has been used to evaluate pulmonary parenchymal changes in PRISm patients, revealing that its pathological changes are primarily characterized by small airway remodeling and microvascular abnormalities, with minimal reductions in pulmonary parenchymal density, distinct from the emphysema-dominant pattern observed in early-to-mid stage COPD [11]. Furthermore, cohort studies reveal that patients with PRISm have higher all-cause mortality rate compared to individuals with normal pulmonary function and experience increase in cardiovascular events, alongside a significantly elevated risk of systemic comorbidities [12–15]. However, clinical recognition of this phenotype remains low and many patients miss the optimal therapeutic window.

Recent advances in CT-based radiomics have enabled the high throughput extraction of quantitative structural and functional imaging features, thereby facilitating more precise phenotyping of respiratory diseases. Most prior studies have focused on single dimensional analyses (e.g., detecting and staging COPD), without fully integrating bronchial and parenchymal changes into a unified framework. In particular, automated, multi-category classification models that distinguish among normal pulmonary function, PRISm, and COPD remain scarce. To address this gap, the present study proposes a three-category classification approach incorporating two complementary radiomic models: one built on quantitative airway tree parameters and another encompassing both parenchymal and airway tree features. This model design aims not only to capture the distinct imaging hallmarks of PRISm but also to enhance early detection and improve risk stratification. By identifying PRISm specific imaging biomarkers and offering robust quantitative decision support tools, our work has the potential to advance both clinical diagnosis and precision management of chronic airway diseases.

## Methods

### Study design and participants

**Fig. 1:**
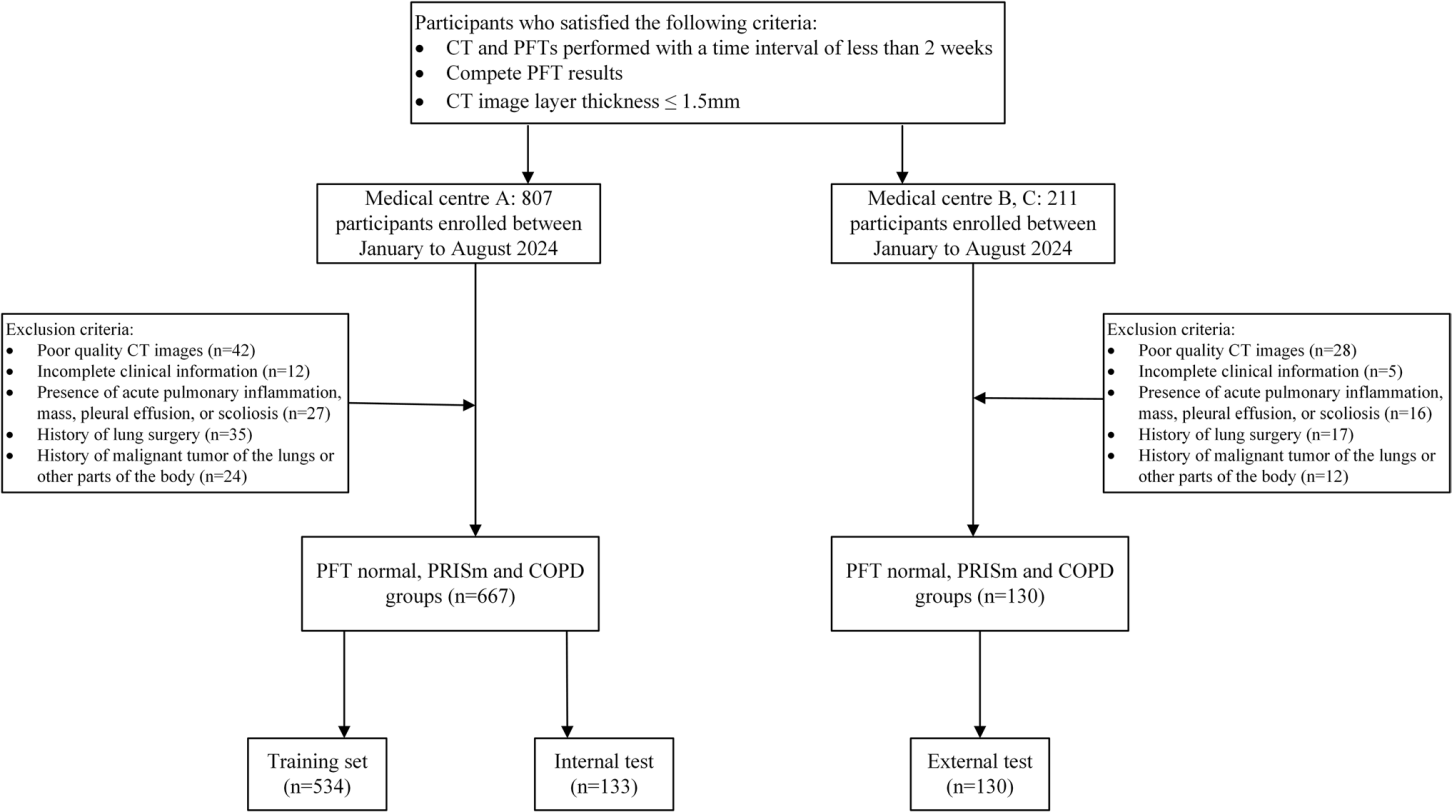
Flowchart showing the participant recruitment process. *Note*. PFT, pulmonary function test; PRISm, preserved ratio impaired spirometry; COPD, chronic obstructive pulmonary disease

This retrospective study was approved by the Ethics Review Committee of our hospital (No.20240123). The requirement for obtaining written informed consent was waived. Patient data were retrospectively and continuously collected from our hospital (Medical Centre A) between January and August 2024. Data from medical center B and medical center C were retrospectively gathered to create an external test set. The inclusion criteria were: (1) completion of both chest CT and pulmonary function tests (PFTs) within 2 weeks; (2) slice thickness ≤ 1.5 mm with acceptable image quality; and (3) complete PFT data. Exclusion criteria included acute pulmonary inflammation, mass, pleural effusion, scoliosis, a history of lung surgery or malignancy, or missing clinical information (Fig. 1). Demographic data such as age, gender, height, weight, body mass index (BMI), surgical history, and malignancy history were recorded for each participant.

### Imaging protocol and pulmonary function tests

Chest CT scans were obtained using one of three systems: GE LightSpeed VCT 64-slice, Siemens Somatom Definition Flash, or United Imaging u760. The scanning parameters were as follows: tube current 80–140 mA, tube voltage 80–120 kV, slice thickness 1–2 mm, and both high resolution and soft tissue reconstruction algorithms in full inspiration phase. Patients were positioned supine with arms elevated to minimize artifacts, and the scan range extended from the thoracic inlet to the lung bases.

Pulmonary function tests followed a standardized protocol. Under physician guidance, each participant performed at least three acceptable maneuvers, and the best result was recorded. FEV₁ and FVC were expressed as percentages of predicted values, calculated using the global lung initiative equations adjusted for age, gender, and height.

### Airway tree and whole lung segmentation

All CT images underwent a preprocessing pipeline comprising: (1) anonymization, (2) resampling using linear interpolation to a uniform voxel dimension of 1 × 1 × 1 mm^3^, and (3) gray level normalization (window range − 1024 to 200 HU). Semi-automated segmentation was performed in Mimics Research (Version 21.0, Materialise, Leuven, Belgium):

For the airway tree, a region growing algorithm based on air density (− 1000 ± 200 HU) was applied starting from the tracheal seed point and extending distally, guided by anatomical knowledge. Three-dimensional reconstructions in the axial, coronal, and sagittal planes were then used for manual correction of missing distal airways or areas affected by artifacts.

**Fig. 2:**
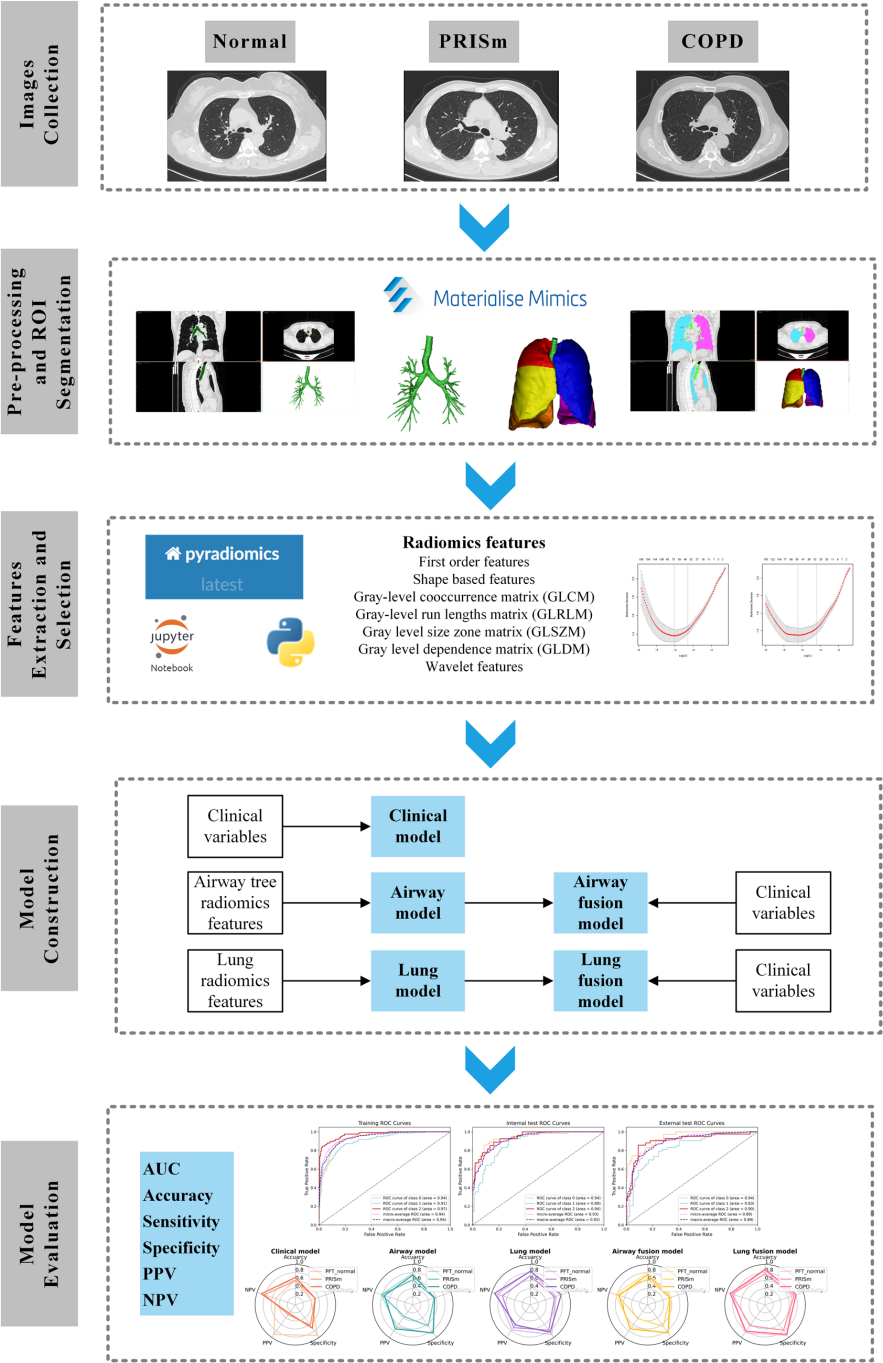
Overview of the framework of the three-category classification models

For the whole lung parenchyma, threshold-based segmentation and morphological smoothing were conducted under the guidance of the bronchial centerline, followed by manual adjustments to remove vessel extensions and pleural adhesions. The resulting 3D models were exported for subsequent analyses (Fig. 2).

### Radiomic feature extraction

Using PyRadiomics (Version 3.0.1, https://readthedocs.org/projects/pyradiomics/), a total of 1218 radiomic features were extracted from each of the two regions of interest (the airway tree and the whole lung), yielding 2436 features in total. These included: (1) first-order statistics (18 features), capturing intensity histogram properties; (2) shape features (14 features), such as volume, surface area, and sphericity; (3) texture features (498 features), derived from gray level cooccurrence matrix, gray level run length matrix, gray level dependence matrix, and gray level size zone matrix; and (4) wavelet transformed features (688 features), providing multi-scale texture and intensity patterns.

Feature selection was conducted in two stages. First, a maximum relevance minimum redundancy (mRMR) algorithm identified the top 100 features most relevant to the classification task while minimizing redundancy. Next, least absolute shrinkage and selection operator (LASSO) regression, optimized via tenfold cross-validation, identified a refined subset of airway tree and lung features under the optimal regularization parameter.

### Model construction

Five multinomial logistic regression models were developed for three-category classification: a clinical model incorporating clinical variables; an airway model based on selected airway tree radiomic features; a lung model utilizing selected whole lung radiomic features; an airway fusion model integrating clinical parameters with airway tree features; and a lung fusion model combining clinical parameters with whole lung features.

### Statistical analysis

All analyses were performed using SPSS software (Version 23.0 IBM SPSS Statistics Software, Chicago, IL, USA) and R software (version 4.3.0; R Foundation for Statistical Computing, Vienna, Austria). Normality of continuous variables was assessed using the Shapiro–Wilk test. For normally distributed continuous variables, data were presented as mean ± standard deviation, whereas non-normally distributed continuous variables were reported as medians with interquartile ranges (P25–P75). Categorical variables were expressed as frequencies (%). Intergroup comparisons used the Kruskal–Wallis rank-sum test (for non-normally distributed data) or the Chi-square test (for categorical data). Models were developed using multinomial logistic regression analysis. Model performance was assessed by accuracy, sensitivity, specificity, positive predictive value, negative predictive value, and the area under the receiver operating characteristic curve (AUC). Delong tests were used to compare AUCs. And a* P*-value < 0.05 was considered statistically significant.

## Results

### Participants characteristic

A total of 1018 participants were initially screened. After applying the inclusion and exclusion criteria, 797 participants (mean age: 62.26 ± 12.65 years; 482 men) were included in the study. These participants were randomly allocated into a training set (*n* = 534; mean age: 61.98 ± 13.24 years; 326 men) and an internal test set (*n* = 133; mean age: 61.97 ± 11.50 years; 88 men) in an 8:2 ratio. An external test set consisting of 130 participants (mean age: 63.72 ± 11.21 years; 68 men) was also established. Among all participants, 43.91% (350/797) exhibited normal pulmonary function, 31.87% (254/797) were classified as having PRISm, and 24.22% (193/797) were diagnosed with COPD. A summary of the clinical variables across all datasets is provided in Table 1.

**Table 1:** Participants information for the development dataset and external test set

Variables	Training set (*n* = 534)			*P*-value	Internal test set (*n* = 133)			*P*-value	External test set (*n* = 130)			*P*-value
	PFT-normal (*n* = 251)	PRISm (*n* = 159)	COPD (*n* = 124)		PFT-normal (*n* = 64)	PRISm (*n* = 42)	COPD (*n* = 27)		PFT-normal (*n* = 35)	PRISm (*n* = 53)	COPD (*n* = 42)	
Age^*^ (years)	55.09 ± 12.39	66.64 ± 12.07	69.95 ± 8.47	< 0.001	55.69 ± 11.63	67.79 ± 7.84	67.81 ± 8.02	< 0.001	53.91 ± 10.18	65.91 ± 9.18	69.14 ± 9.20	< 0.001
Gender^§^				< 0.001				0.025				0.002
Female	129 (51.39%)	59 (37.11%)	20 (16.13%)		28 (43.75%)	13 (30.95%)	4 (14.81%)		24 (68.57%)	26 (49.06%)	12 (28.57%)	
Male	122 (48.61%)	100 (62.89%)	104 (83.87%)		36 (56.25%)	29 (69.05%)	23 (85.19%)		11 (31.43%)	27 (50.94%)	30 (71.43%)	
Height (cm)	165 (158.5–171.5)	167 (161–173)	170 (165.5–174.5)	0.071	167.5 (161.5–173.5)	166 (160.5–171.5)	170 (166.5–173.5)	0.414	160 (152.5–167.5)	162 (155–169)	164 (156.5–170.5)	0.867
Weight (kg)	66 (58–74)	65 (59–71)	65 (57.5–72.5)	0.018	66 (58–74)	64.5 (57–72)	64 (57–71)	0.328	63 (56–70)	69 (58–80)	70 (59–81)	0.516
BMI (kg/m^2^)	24.03 (22.13–26.03)	23.45 (21.42–25.48)	23.19 (21.2–25.18)	< 0.001	23.94 (21.98–25.89)	23.59 (21.44–25.74)	21.63 (19.07–24.19)	0.088	23.89 (21.43–26.35)	24.65 (21.1–28.2)	25.20 (21.85–28.55)	0.043

*Data are presented as mean ± SD. ^§^Data represent the number of participants, with data in parentheses representing percentages. Other data represent the median, with data in parentheses representing the interquartile range. *PFT* pulmonary function test, *PRISm* preserved ratio impaired spirometry, *COPD* chronic obstructive pulmonary disease

In the training set, univariate analysis indicated that age, gender, weight, and BMI differed significantly among the three groups (all* P* < 0.05). Subsequent multinomial logistic regression revealed that age [odds ratio (OR) 1.08; 95% confidence interval (CI) 1.07–1.10; *P* < 0.001], gender [OR 2.25; 95%CI 1.48–3.42; *P* < 0.001], and BMI [OR 0.91; 95%CI 0.86–0.96; *P* < 0.001] were independent predictors of pulmonary function. These factors were then incorporated into the clinical model (Table S1).

### Feature selection

During feature selection, the mRMR algorithm was first used to filter radiomic features extracted from both the airway tree and the whole lung. From a total of 2436 features (1218 airway tree features and 1218 whole lung features), the top 100 features most relevant to the classification task and with the lowest redundancy were identified. These 100 candidate features were then entered into LASSO regression model with tenfold cross-validation to determine the optimal regularization parameter. By shrinking coefficients of less informative features, LASSO ultimately selected 35 airway tree radiomic features and 48 whole lung radiomic features for final model construction (Table S2).

### Model performance

Five classification models were developed and evaluated on the training, internal test, and external test sets. Across all datasets, the lung fusion model, which integrates clinical and whole lung radiomic features, achieved the best performance (Tables 2, 3). In the training set, the lung fusion model achieved AUCs of 0.935 (95% CI 0.915–0.955), 0.905 (95% CI 0.878–0.933), and 0.972 (95% CI 0.958–0.986) for the three classification categories, respectively. These values were significantly higher than those of the clinical model (AUCs 0.812, 0.658, 0.777) and the airway fusion model (AUCs 0.910, 0.843, 0.965), with statistical significance at *P* < 0.001 and *P* = 0.004.

**Table 2:** The areas under the receiver operating characteristic curves of the five models in the training set, the internal test set, and the external test set

Parameter	PFT-normal	PRISm	COPD
Clinical model			
Training set	0.812 (0.776–0.848)	0.658 (0.609–0.707)	0.777 (0.735–0.820)
Internal test set	0.813 (0.740–0.885)	0.649 (0.562–0.706)	0.751 (0.645–0.856)
External test set	0.811 (0.727–0.895)	0.594 (0.496–0.692)	0.710 (0.620–0.800)
Airway model			
Training set	0.869 (0.840–0.899)	0.782 (0.738–0.825)	0.953 (0.935–0.971)
Internal test set	0.853 (0.789–0.918)	0.842 (0.775–0.909)	0.895 (0.824–0.967)
External test set	0.840 (0.765–0.915)	0.703 (0.612–0.793)	0.832 (0.759–0.905)
Lung model			
Training set	0.909 (0.885–0.933)	0.860 (0.826–0.894)	0.960 (0.941–0.980)
Internal test set	0.905 (0.849–0.952)	0.856 (0.787–0.924)	0.928 (0.871–0.985)
External test set	0.897 (0.837–0.956)	0.782 (0.702–0.862)	0.906 (0.846–0.965)
Airway fusion model			
Training set	0.910 (0.886–0.934)	0.843 (0.805–0.881)	0.965 (0.950–0.980)
Internal test set	0.908 (0.860–0.956)	0.877 (0.816–0.938)	0.928 (0.871–0.985)
External test set	0.870 (0.802–0.938)	0.737 (0.651–0.823)	0.819 (0.741–0.897)
Lung fusion model			
Training set	0.935 (0.915–0.955)	0.905 (0.878–0.933)	0.972 (0.958–0.986)
Internal test set	0.939 (0.900–0.978)	0.886 (0.831–0.940)	0.940 (0.896–0.984)
External test set	0.939 (0.898–0.979)	0.830 (0.758–0.902)	0.904 (0.841–0.966)

Data are presented as AUC, with 95% confidence intervals in parentheses

**Table 3:** Diagnostic performance of lung fusion model for predicting the pulmonary function state

Parameter	AUC^*^ (95%CI)	Accuracy	Sensitivity	Specificity	PPV	NPV
Training set						
PFT-normal	0.935 (0.915–0.955)	86.14 (460/534)	83.15 (222/267)	89.14 (238/267)	88.45 (222/251)	84.10 (238/283)
PRISm	0.905 (0.878–0.933)	85.58 (457/534)	78.08 (114/146)	88.40 (343/388)	71.70 (114/159)	91.47 (343/375)
COPD	0.972 (0.958–0.986)	93.82 (501/534)	87.60 (106/121)	95.64 (395/413)	85.48 (106/124)	96.34 (395/410)
Internal test set						
PFT-normal	0.939 (0.900–0.978)	86.47 (115/133)	84.85 (56/66)	88.06 (59/67)	87.50 (56/64)	85.51 (59/69)
PRISm	0.886 (0.831–0.940)	80.45 (107/133)	73.53 (25/34)	82.83 (82/99)	59.52 (25/59)	90.11 (82/91)
COPD	0.940 (0.896–0.984)	87.97 (117/133)	66.67 (22/33)	95.00 (95/100)	81.48 (22/27)	89.62 (95/106)
External test set						
PFT-normal	0.939 (0.898–0.979)	86.15 (112/130)	69.77 (30/43)	94.25 (82/87)	85.71 (30/35)	86.32 (82/95)
PRISm	0.830 (0.758–0.902)	76.92 (100/130)	75.56 (34/45)	77.65 (66/85)	64.15 (34/53)	85.71 (66/77)
COPD	0.904 (0.841–0.966)	87.69 (114/130)	80.95 (34/42)	90.91 (80/88)	80.95 (34/42)	90.91 (80/88)

*Data are presented as AUC, with 95% confidence intervals in parentheses. Other data are percentages; with data in parentheses representing the numerators/denominators. *AUC* area under the curve, *NPV* negative predictive value, *PPV* positive predictive value

**Fig. 3:**
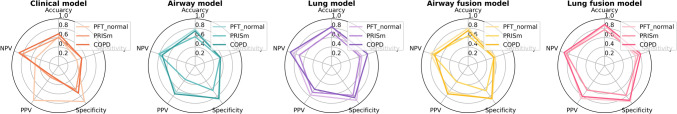
Radar maps of the five diagnostic indexes (accuracy, sensitivity, specificity, PPV, and NPV) in the external test set for all five models. *Note*. NPV, negative predictive value; PPV, positive predictive value

**Fig. 4:**
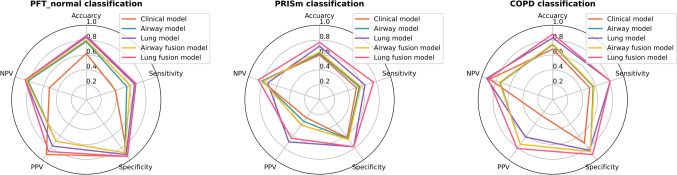
Radar maps of the five diagnostic indexes for the three-category classification of pulmonary function in the external test set

**Fig. 5:**
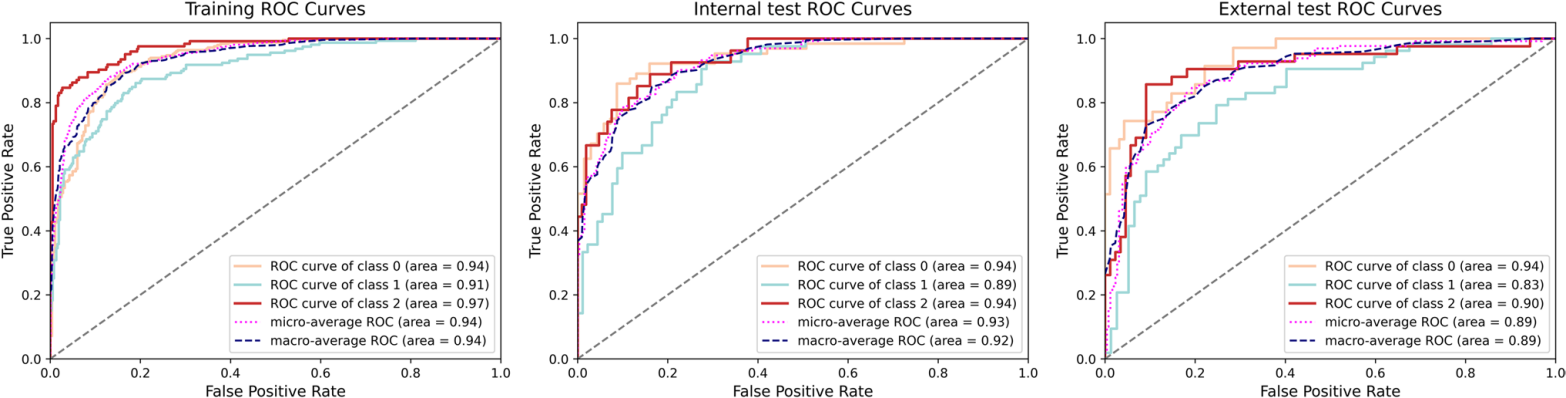
Diagnostic performance of the lung fusion model. a Performance in the training set; b performance in the internal test set; c performance in the external test set. Note. Class 0 represents PFT-normal, Class 1 represents PRISm, and Class 2 represents COPD

In the internal test set, the lung fusion model continued to demonstrate superior performance, achieving AUCs of 0.939, 0.886, and 0.940, with corresponding accuracies of 86.47%, 80.45%, and 87.97%. In the external test set, this model also outperformed others, yielding the highest AUCs (0.939, 0.830, and 0.904) and accuracies (86.15%, 76.92%, and 87.69%), surpassing the airway fusion model in terms of accuracy, sensitivity, and specificity (Table S3, Figs. 3, 4, 5). As shown in Fig. 4, all models exhibited better classification performance for the PFT-normal and COPD groups compared to the PRISm group. Specifically, when predicting the PRISm group, the lung fusion model, which demonstrated the best overall performance among all models, achieved an AUC of 0.830, an accuracy of 76.92%, a sensitivity of 75.56%, and a specificity of 77.65%. Despite the smaller sample size and class imbalance of the PRISm group, the model still exhibited relatively robust performance.

## Discussion

PRISm and COPD not only lead to progressive declines in pulmonary function but also substantially increase the risk of cardiovascular diseases, depression, anxiety, cognitive impairment, and gout, making early detection and intervention crucial for improving patient outcomes [12–14, 16, 17]. In this study, we constructed two novel three-category classification models based on clinical variables and radiomic features extracted from both the airway tree and the whole lung. Our results demonstrate that the lung fusion model, which integrates age, gender, and BMI with whole lung radiomic features, consistently outperforms other models across the training, internal test, and external test sets. This superior performance underscores the added value of quantitative imaging data from the lung parenchyma in accurately distinguishing PRISm and COPD—two conditions that, despite overlapping clinical presentations, require distinct management approaches. By incorporating CT-based radiomic analysis into clinical workflows, clinicians may be able to identify pulmonary abnormalities earlier, enabling timely intervention and potentially improving patient outcomes. Moreover, radiomics-based quantification of parenchymal abnormalities and small airway damage could facilitate personalized treatment planning, supporting more precise and individualized disease management.

With the increasing application of CT in lung cancer screening and the rapid advancement of neural network methods, recent studies have shifted toward analyzing changes across the entire lung. For example, Deng et al. employed both nodule-based and whole lung radiomics to distinguish benign from malignant lung nodules [18], while other researchers used tumor based and whole lung radiomic approaches to assess postoperative survival in patients with non-small cell lung cancer [19, 20]. These approaches underscore the value of comprehensive imaging when characterizing a broad range of pathological alterations. PRISm and COPD are likewise heterogeneous conditions involving small airway remodeling, pulmonary vasculature changes, and emphysema formation [2, 14, 21, 22]. Although they are commonly diagnosed using pulmonary function tests, early stages of tissue damage can yield “pseudo-normal” ventilatory measurements, thereby delaying timely detection [23, 24]. Leveraging low-dose CT to address these limitations, Park et al. developed two convolutional neural network models to predict FVC and FEV1 from three-dimensional features extracted from a series of coronal low-dose CT images, reporting concordance correlation coefficients of 0.94 and 0.91, respectively [25]. Their findings demonstrated the feasibility of low-dose CT for pulmonary function prediction and paved the way for imaging-based approaches that enable earlier and more precise diagnosis of PRISm and COPD.

Subsequently, Zhou et al. proposed a nomogram based on whole lung radiomic features for COPD diagnosis (AUC = 0.893, 0.873, 0.853 in training, internal, and external cohorts, respectively). This approach demonstrates that whole lung radiomics is a robust biomarker for COPD in both lung cancer screening and routine CT. The intuitive nomogram aids early detection and reduces underdiagnosis in clinical practice [26]. Zhou et al. subsequently introduced a radiomics nomogram incorporating clinical factors to identify PRISm, achieving AUCs of 0.787, 0.773, and 0.702 in the training, internal, and external cohorts, respectively, thereby outperforming the radiomics signature alone and a purely clinical factor model [27]. However, these studies focused only on the left and right lungs and did not consider the main bronchi and their subdivisions, although airway pathologies such as bronchiectasis and airway wall thickening are common in COPD. To address these limitations, we initially examined radiomic features of the trachea, bilateral main bronchi, and their branches, and then extended our analysis to the entire lung parenchyma. We developed two ordinal three-category models to simultaneously differentiate PRISm and COPD from normal individuals; this whole lung integrated approach demonstrated superior predictive performance. Compared with the clinical model, both the airway fusion model and the lung fusion model achieved significantly better classification across all datasets (*P* < 0.05), suggesting that radiomic features extracted from the airway tree and the whole lung add important diagnostic information. Furthermore, the lung fusion model outperformed the airway fusion model in both internal and external test sets, likely because whole lung radiomics captures a broader range of structural changes that include vascular and interstitial alterations, thus offering a more comprehensive quantitative assessment of disease-related imaging abnormalities. In contrast, accurate extraction of airway tree features is strongly influenced by image quality and segmentation algorithms, emphasizing the need for continued methodological refinement in future research.

The model incorporated age, sex, and BMI as clinical factors, consistent with previous findings that PRISm is more prevalent in older adults and women, and that BMI correlates with early declines in pulmonary function [9, 10]. Among the most influential radiomic features identified by the lung fusion model, three reflected alterations in lung texture and density, indicating structural complexity and functional compromise. Specifically, wavelet LLH gldm dependence entropy represents parenchymal heterogeneity, with higher values signifying more severe structural abnormalities; log sigma 5 0 mm 3D first-order maximum highlights the highest-intensity voxels following edge enhancement, potentially capturing fibrotic or air-trapping regions; and wavelet LLL first-order 10Percentile indicates low-density areas suggestive of emphysematous changes. These findings collectively underscore the practical value of whole lung radiomics in detecting subtle functional differences and suggest that it may serve as a quantitative imaging biomarker for the early detection and classification of chronic airway diseases.

Although the results of this study are encouraging, the model exhibited lower accuracy in identifying PRISm patients compared to COPD patients. Several factors may account for this discrepancy. First, the relatively smaller sample size of the PRISm group, compared with the normal groups, may have limited the model’s ability to fully learn and accurately differentiate radiomic features specific to PRISm. Second, PRISm itself is a highly heterogeneous clinical condition, its early pathological alterations often present as subtle and diffuse imaging findings, unlike COPD, which typically exhibits more obvious emphysematous changes or airway wall thickening [4, 11]. These characteristics increase the difficulty of model-based discrimination. To enhance the model’s ability to identify PRISm, future studies should expand the sample size of PRISm patients, incorporate more detailed clinical indicators (such as pulmonary function parameters and biomarkers), and utilize longitudinal imaging data to better capture disease progression and improve model generalizability. In addition, as a multicenter retrospective study, this work encountered issues with uneven data distribution among centers. Most cases originated from a single center, while the external validation cohort was relatively small. This imbalance may have contributed to model overfitting and limited the accurate assessment of model generalizability. Although multiple evaluation metrics indicated good model performance, future studies should aim to include more participating centers, expand external validation datasets, and standardize imaging acquisition protocols across sites to strengthen external validation.

Finally, despite efforts to perform image resampling and normalization, variations in scanning protocols and imaging equipment among centers may still have affected the results. Furthermore, patients with pulmonary masses were excluded to avoid interference with radiomic feature extraction, which may limit the applicability of the model in more complex clinical scenarios. Nevertheless, this study provides important preliminary evidence and methodological foundations for future large-scale, prospective multicenter research.

## Conclusions

This study developed several three-category predictive models combining CT-based radiomics and clinical variables to distinguish normal pulmonary function, PRISm, and COPD. The lung fusion model showed the best performance, emphasizing the role of imaging biomarkers in early assessment. It may enable earlier detection of pulmonary abnormalities before significant functional decline, supporting timely intervention and improved outcomes. In addition, this model could be integrated into low-dose CT lung cancer screening programs to enable simultaneous screening for both lung cancer and chronic airway diseases.

## Supplementary Information

Below is the link to the electronic supplementary material.

Supplementary file1 (DOCX 24 KB)

## Data Availability

The datasets used or analysed during the current study are available from the corresponding author on reasonable request.

## Abbreviations

AUC: Area under the curve
BMI: Body mass index
CI: Confidence interval
COPD: Chronic obstructive pulmonary disease
CT: Computed tomography
FEV1: Forced expiratory volume in one second
FVC: Forced vital capacity
LASSO: Least absolute shrinkage and selection operator
mRMR: Maximal redundancy minimal relevance
OR: Odds ratio
PFT: Pulmonary function test
PRISm: Preserved ratio impaired spirometry
