# Supplementary material for: Chest CT imaging for differentiating normal, PRISm, and COPD in comparison with pulmonary function tests
Source: Radiol Med. 2025 Aug 21;130(11):1786–96. doi: 10.1007/s11547-025-02061-4 (PMC12605617; doi:10.1007/s11547-025-02061-4)
Supplement: Supplementary file 1 — Supplementary file1 (DOCX 24 KB) [file 11547_2025_2061_MOESM1_ESM.docx]

**Supplemental material**

Table S1: Univariable and multivariate analysis for clinical variables.

Table S2: Selected radiomics features and their categories.

Table S3: Comparison of performance of the five models in the external test set.

This supplemental material was provided by the authors to provide readers with additional information on their work.

**Table S1: Univariable and multivariate analysis for clinical variables.**

| Parameter | Univariate analysis | | | Multivariate analysis | | |
| --- | --- | --- | --- | --- | --- | --- |
|  | OR | 95%CI | *P*-value | OR | 95%CI | *P*-value |
| Age (years) | 1.09 | 1.07-1.11 | ＜0.001 | 1.08 | 1.07-1.10 | ＜0.001 |
| Gender | 3.05 | 2.17-4.30 | ＜0.001 | 2.25 | 1.48-3.42 | ＜0.001 |
| Height | 1.02 | 1.00-1.04 | 0.1 |  |  |  |
| Weight | 0.98 | 0.97-0.99 | 0.006 | 0.99 | 0.97-1.02 | 0.58 |
| BMI (Kg/m^2^) | 0.91 | 0.86-0.95 | ＜0.001 | 0.91 | 0.86-0.96 | ＜0.001 |

Note. OR: Odds ratio; CI: Confidence interval.

**Table S2: Selected radiomics features and their categories.**

| Feature category | Airway tree features (n = 35) | Lung features (n = 48) | Description |
| --- | --- | --- | --- |
| First order statistical features | 10 | 6 | Histogram features, such as mean, standard deviation, kurtosis, etc. |
| Shape features | 4 | 5 | Volume, surface area, sphericity, etc. |
| Gray level cooccurrence matrix features | 6 | 3 | Contrast, homogeneity, etc. |
| Gray level dependence matrix features | 3 | 2 | Non-Uniformity, dependence Entropy, etc |
| Gray level run length matrix features | 1 | 2 | Short/long-run emphasis, etc. |
| Gray level size zone matrix features | 4 | 7 | Zone entropy, non-uniformity, etc. |
| Wavelet transformed features | 7 | 23 | Statistical and texture features derived from wavelet transformation |

**Table S3: Comparison of performance of the five models in the external test set.**

| Parameter | AUC^*^ | | | Accuracy | | | Sensitivity | | | Specificity | | | PPV | | | | NPV | | |
| --- | --- | --- | --- | --- | --- | --- | --- | --- | --- | --- | --- | --- | --- | --- | --- | --- | --- | --- | --- |
|  | PFT-normal | PRISm | COPD | PFT-normal | PRISm | COPD | PFT-normal | PRISm | COPD | PFT-normal | PRISm | COPD | PFT-normal | PRISm | COPD | PFT-normal | | PRISm | COPD |
| Clinical model | 0.811 (0.727-0.895) | 0.594 (0.496-0.692) | 0.710 (0.620-0.800) | 62.31 (81/130) | 60.00 (78/130) | 68.46 (89/130) | 41.03 (32/78) | 51.61 (16/31) | 52.38 (11/21) | 94.23 (49/52) | 62.63 (62/99) | 71.56 (78/109) | 91.43 (32/35) | 30.19 (16/53) | 26.19 (11/42) | 51.58 (49/95) | | 80.52 (62/77) | 88.64 (78/88) |
| Airway model | 0.840 (0.765-0.915) | 0.703 (0.612-0.793) | 0.832 (0.759-0.905) | 77.69 (101/130) | 62.31 (81/130) | 73.85 (96/130) | 57.14 (24/42) | 55.88 (19/34) | 57.41 (31/54) | 87.50 (77/88) | 64.58 (62/96) | 85.53 (65/76) | 68.57 (24/35) | 35.85 (19/53) | 73.81 (31/42) | 81.53 (77/95) | | 80.52 (62/77) | 73.86 (65/88) |
| Lung model | 0.897 (0.837-0.956) | 0.782 (0.702-0.862) | 0.906 (0.846-0.965) | 83.85 (109/130) | 71.54 (93/130) | 83.08 (108/130) | 67.50 (27/40) | 63.79 (37/58) | 81.25 (26/32) | 91.11 (82/90) | 77.78 (56/72) | 83.67 (82/98) | 77.14 (27/35) | 69.81 (37/53) | 61.90 (26/42) | 86.32 (82/95) | | 72.73 (56/77) | 93.18 (82/88) |
| Airway fusion model | 0.870 (0.802-0.938) | 0.737 (0.651-0.823) | 0.819 (0.741-0.897) | 80.00 (104/130) | 63.85 (83/130) | 74.62 (97/130) | 61.54 (24/39) | 57.89 (22/38) | 58.49 (31/53) | 87.91 (80/91) | 66.30 (61/92) | 85.71 (66/77) | 68.57 (24/35) | 41.51 (22/53) | 73.81 (31/42) | 84.21 (80/95) | | 79.22 (61/77) | 75.00 (66/88) |
| Lung fusion model | 0.939 (0.898-0.979) | 0.830 (0.758-0.902) | 0.904 (0.841-0.966) | 86.15 (112/130) | 76.92 (100/130) | 87.69 (114/130) | 69.77 (30/43) | 75.56 (34/45) | 80.95 (34/42) | 94.25 (82/87) | 77.65 (66/85) | 90.91 (80/88) | 85.71 (30/35) | 64.15 (34/53) | 80.95 (34/42) | 86.32 (82/95) | | 85.71 (66/77) | 90.91 (80/88) |

Note. *Data are presented as AUC, with 95% confidence intervals in parentheses. Other data are percentages; with data in parentheses representing the numerators/denominators. PFT, pulmonary function test; PRISm, preserved-ratio impaired spirometry; COPD, chronic obstructive pulmonary disease.; AUC, area under the curve. NPV, negative-predictive value; PPV, positive-predictive value.
